# Fencing Large Predator-Free and Competitor-Free Landscapes for the Recovery of Woodland Caribou in Western Alberta: An Ineffective Conservation Option

**DOI:** 10.3390/ani7010002

**Published:** 2016-12-28

**Authors:** Gilbert Proulx, Ryan K. Brook

**Affiliations:** 1 Alpha Wildlife Research & Management Ltd., Sherwood Park, AB T8H 1W3, Canada; 2 Department of Zoology and Entomology, University of Fort Hare, Private Bag X1314, Alice 5700, South Africa; 3 Department of Animal and Poultry Science & the Indigenous Land Management Institute, University of Saskatchewan, Saskatoon, SK S7N5A8, Canada; ryan.brook@usask.ca

**Keywords:** caribou, wolf, fencing, control, wildlife management

## Abstract

**Simple Summary:**

In western Alberta, Canada, in order to recover the Little Smoky boreal woodland caribou (*Rangifer tarandus*) population, the provincial government announced a plan in June 2016 to create a 100-km^2^-fenced enclosure that would encompass part of the caribou population range. Within the enclosure, all predators and other ungulates will be killed. The fenced area will be dedicated entirely to the farming of caribou, with the intent of releasing weaned calves into adjacent areas with continued intensive wolf (*Canis lupus*) control throughout the region. Industrial activities will be allowed to continue within the enclosure. In this review, we assess the government’s proposed program on the basis of questions related to the long-term recovery and sustainability of the caribou population, and the conservation and welfare of wildlife populations and individuals. We conclude that this program is unlikely to safeguard the future of this caribou population, will jeopardize wildlife communities inside and outside the fenced enclosure, and will cause harm to wild populations and individual animals. We recommend an alternative habitat conservation program which is ecologically justified over the long term.

**Abstract:**

In Canada, boreal woodland caribou (*Rangifer tarandus caribou*) are listed as “threatened” throughout their range due directly and indirectly to habitat loss. In western Alberta, in order to recover the Little Smoky boreal caribou population, the provincial government announced a plan to create a 100-km^2^-fenced enclosure that would encompass part of the caribou population range. Within the enclosure, all predators and other ungulate species will be killed. The fenced area will be dedicated entirely to the farming of caribou, with the intent of releasing weaned calves into adjacent areas with continued intensive wolf (*Canis lupus*) killing throughout the region. Industrial activities will be allowed to continue within the enclosure. In this review, we assess the government’s proposed program on the basis of questions related to the long-term recovery and sustainability of the caribou population, and the conservation and welfare of wildlife populations and individuals. We conclude that this program is unlikely to safeguard the future of this caribou population, will jeopardize wildlife communities inside and outside the fenced enclosure, and will cause harm to wild populations and individual animals. We recommend an alternative habitat conservation program which is ecologically justified over the long term, and invite the scientific community to object to the implementation of the government’s proposed Little Smoky caribou recovery program.

## 1. Introduction

In Canada, boreal woodland caribou (*Rangifer tarandus caribou*) are listed as “threatened” throughout their range, i.e., they are likely to become endangered if nothing is done to reverse the factors leading to their extirpation or extinction, under Canada’s Species at Risk Act [1,2]. In western Alberta, in order to recover the Little Smoky (LS) boreal woodland caribou population, the provincial government announced a plan in June 2016 to create a 100-km^2^-fenced enclosure [3] that would encompass <10% of the estimated caribou population range [4], which is largely uninhabited by caribou [5]. Within the enclosure, wolves (*Canis lupus*), bears (*Ursus* spp.), cougars (*Felis concolor),* moose (*Alces alces*), elk (*Cervus canadensis*), and deer (*Odoicoileus* spp.) will be killed to create a predator-free landscape without competitors [3,6]. The fenced area will be dedicated entirely to the farming of caribou, with the intent of releasing weaned calves out of the fenced area into adjacent areas with continued wolf control (Appendix A). The fencing program will cost approximately 15 million Canadian dollars over 10 years [6].

Oil and gas exploitation will be allowed to continue within the enclosure based on a potential voluntary re-scheduling of some activities. Logging will also be permitted (Appendix A). Some seismic lines will be restored slowly over time to minimize access and increase the amount of “undisturbed” habitat, in an effort to meet Environment Canada’s [1] boreal caribou recovery strategy of a minimum of 65% undisturbed habitat within boreal woodland caribou range.

We are aware of only one documented case of farming previously free-ranging caribou in Alberta [7]. In 2006, researchers captured 10 pregnant females from the LS population, and held them in a 4-ha pen within the LS range. One farmed calf died in the enclosure. The other 9 calves were radio-collared and released when they were ≥3 week old. Their survival was compared to that of 7 free-ranging calves. Results were not clear, in part due to the small sample sizes and also because wolf control was implemented in the study area at the same time. The penned calves also habituated to the staff working on the program. Survival of the free-ranging calves was actually higher (71% survival) than that of the farmed calves (50% mortality due to bears and unknown reasons). The cost at the time was estimated to be 40,000 CAD dollars/penned calf. In 2012, a workshop was organized by Oil Sands Leadership Initiative Land Stewardship Working Group suggesting that, in spite of the 2006 failed caribou farming experiment, a large fenced predator-free enclosure would be technically feasible, although challenging and costly to implement [8].

The ultimate reason for the decline of the LS caribou population is habitat alteration (loss, degradation, and/or fragmentation) over 95% of the range due to industrial activities and infrastructure [1]. From 2005 to 2012, rather than executing a habitat conservation program, the Alberta Government implemented widespread and intensive killing of wolves as essentially the only provincial strategy to recover the LS caribou population [9]. However, this program proved to be unsuccessful, as the number of caribou and the annual growth rate for the adult female component remained the same as before the wolf control program [10], and was judged unacceptable for having used inhumane and indiscriminate killing methods that failed to follow standards of the Canadian Council on Animal Care [11]. Removing wolf breeders may actually result in a subdivision of existing wolf territories and an increase in wolf densities locally through compensatory reproduction and colonization [12,13,14].

While there are no studies showing that wolves critically suppress growth of the LS caribou population, it has been established that only 20% of the caribou range met the late-winter habitat requirements of the species [5]. Boreal woodland caribou movements between late-winter and calving ranges are relatively short so that seasonal home ranges overlap [15]; spring habitat during the calving season is likely in short supply as well given the widespread loss of all habitat types. The carrying capacity of the highly fragmented landscape for boreal woodland caribou is very low [5], and wolf culling alone will not protect LS population from extirpation.

The proposed predator-free and competitor-free caribou enclosure has raised concerns among environmental groups [16,17] as well as the scientific community [6]. The purpose of this article is to assess the government’s proposed caribou farming program on the basis of 5 questions:

(1) Will the proposed predator-free and competitor-free enclosure likely safeguard the long-term recovery of the LS caribou population?
(2) Will the caribou farmed in an enclosure likely contribute to the sustainability of the entire LS population?
(3) Will the conservation of wild populations likely be safeguarded?
(4) Will the welfare of individuals likely be safeguarded?
(5) Is there a more effective alternative approach to the proposed predator-free and competitor-free fencing program?

The government’s proposed program appears to be developed within the context of the North American Model of Wildlife Management, which has often focused on specific, human-related management goals [18]. One important criticism of the North American Model is that it is fundamentally based on killing animals at its core, but emphasizes the vague notion that wildlife should only be killed for a ‘legitimate purpose’ [19]. As such, we evaluated the government’s proposed program using a pragmatic view, evaluating the components of the proposed project against existing ecological criteria to evaluate implications of killing the predators and competitors of the boreal woodland caribou, specific approaches used for killing animals, and broader impacts on food webs.

## 2. Will the Proposed Predator-Free and Competitor-Free Enclosure Likely Safeguard the Long-Term Recovery of the LS Caribou Population?

In the LS range, caribou late-winter habitats consist of muskegs with <90% black spruce (*Picea mariana*), often mixed with tamarack (*Larix laricina*) and/or lodgepole pine (*Pinus contorta*), and adjacent upland lodgepole pine stands mixed with other conifers. These habitats that are used by caribou amount to 20% of the overall LS population range [5]. Although caribou range is naturally very heterogenous, this species is associated with large tracts of mature to old, low productivity upland and peatland (muskeg) conifer-dominated forests rich in lichens [4,20]. More than 95% of the LS range has been disturbed due to anthropogenic activities [1]. Habitat loss is the dominant critical factor affecting caribou, and conservation efforts should be largely focused on the protection of the remaining caribou habitat, the establishment of movement corridor networks between suitable muskegs, and habitat restoration. Although the government’s proposed plan intends to restore a certain amount of seismic lines, most of it will be done to reduce access by predators and humans, even though both may travel through forests to reach any region of the range.

While restoration of seismic lines is aimed at blocking access to predators, the overall carrying capacity of the range must be recovered in order to have any chance of long-term population sustainability. In the absence of a comprehensive plan to conserve and connect current caribou habitats, and to transform unusable muskegs into valuable habitats consisting of lichen-bearing tamarack-black spruce stands [5], the government’s proposed caribou conservation program is unlikely to support the long-term recovery of the LS caribou population. Although the proposed enclosure represents less than 10% of the caribou population’s potential range, it will include the bulk of the less fragmented habitats used by caribou in late-winter and likely during the calving season [5,21]. The impact of the enclosure on the carrying capacity of the overall caribou range will be significant, especially given the stated intentions of the provincial government and industry to continue activities that destroy, disturb, and fragment critical woodland caribou habitat.

## 3. Will the Caribou Farmed in an Enclosure Likely Contribute to the Sustainability of the LS Population? 

Farming caribou in predator-free fenced landscapes may simply be a politically expedient approach to simulate a population recovery effort when largely inadequate efforts are being made to conserve existing caribou habitat and better control industrial activities in the LS range [22]. Single species management is widely considered an outdated approach [23]. The proposed fencing project is based on killing predators and prey, despite that the peer-reviewed literature clearly indicates that protection and recovery of caribou habitat is the only sustainable solution [2] and provides scant evidence that predator control is effective. Such a fencing program surely fails to meet any of the specific objectives of the federal woodland caribou recovery plan over the short or long term [1], which is focused on maintaining self-sustaining free-ranging caribou populations. In the absence of effective habitat caribou conservation programs, releasing caribou calves raised in enclosures is unsustainable.

Herbivores born and raised in enclosures without predators are naïve and less sensitive to cues that signify the presence of dangerous carnivores [24]. Although naïve prey have the capacity to process information about predators swiftly, in a single generation in the case of moose [25], the learning process likely varies with situations and species, and is more limited in young animals. For example, where both young and adults are killed rapidly, opportunities for a population for learning will be diminished and possibilities for local extirpation increased [25,26]. Caribou confined to the same areas for some time could have higher levels of parasites and diseases [27,28], and they may not be healthy representatives of a wild population. Introducing confined animals back into the wild can create new risks to the free-ranging population. Risks of caribou calves being conditioned to humans is an important concern.

## 4. Will the Conservation of Wild Populations Likely Be Safeguarded?

Farming caribou at the expense of whole wildlife communities and ecosystems is a false conservation effort and is contradictory to the notion of ecosystem management. This program fails to comply with the Canadian Species at Risk Act designed to meet one of Canada’s key commitments under the International Convention on Biological Diversity. The purposes of the Act are to prevent Canadian indigenous species, subspecies, and distinct populations from becoming extirpated or extinct, to provide for the recovery of endangered or threatened species, and encourage the management of other species to prevent them from becoming at risk [29]. In the LS range, the grizzly bear (*Ursus arctos*) is a provincially “threatened” species, and the wolverine (*Gulo gulo*) is classified as “may be at risk”. 

Large carnivores deliver economic and ecosystem services via direct and indirect pathways that help maintain mammal, avian, invertebrate, and herpetofauna abundance, richness and health. Further, they affect other ecosystem processes and conditions, such as scavenger subsidies, disease dynamics, carbon storage, stream morphology, and crop production [30]. Maintaining large-scale, predator-free fenced areas and ongoing killing of predators outside the enclosures results in the creation of truncated wildlife communities that are ecologically depauperate. 

**Figure 1:**
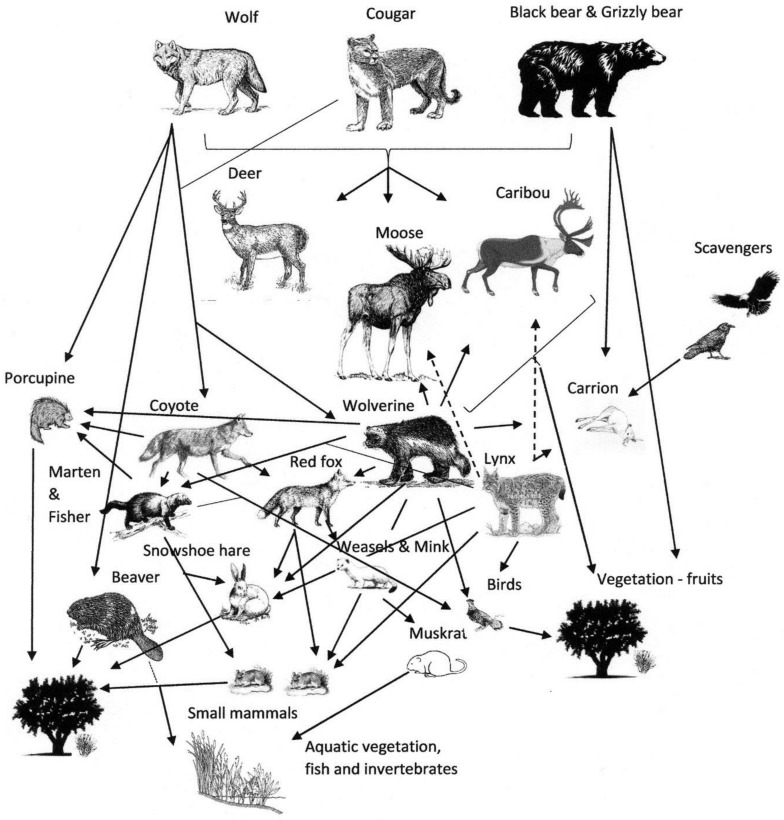
Conceptual diagram of a relatively complex food web in the Little Smoky caribou population range, Alberta, Canada. The proposed fencing and predator control efforts will likely have dramatic impacts on this already human-modified food web.

Fenced wildlife communities experience top-down and bottom-up ecological effects [31,32,33,34], this resulting in a predator-herbivore-vegetation disequilibrium. In the LS caribou range, the proposed predator-free and competitor-free enclosure will affect a complex food web (Figure 1), curtail the dispersal movements of many species, and may jeopardize the dynamics and genetic flow of wildlife populations that are found inside and adjacent to the enclosure, in an already highly modified landscape.

## 5. Will the Welfare of Individuals Likely Be Safeguarded?

Fencing has animal welfare implications, as it may prevent escape of animals from forest fires [35] and modify animal social systems. Animal welfare concerns will be apparent at the individual animal level. Ungulates will be culled through a combination of ungulate harvest by Indigenous peoples, and general and special hunting license opportunities [3]. In Alberta, the culling of predators is achieved with the use of aerial shooting, killing neck snares, and poisoning, methods that all fail to meet Canadian animal welfare standards and because they cause unnecessary pain and suffering, have been criticized extensively by conservation groups and scientists [11,36]. Shooting a running animal from a moving helicopter is prone to error and not conducive to shots that quickly and consistently render animals insensitive to pain or produce a consistently quick kill [11]. Strychnine causes long painful deaths [37], and its use for euthanasia is unacceptable under any circumstances by both government agencies and veterinary organizations [33]. Furthermore, it is an indiscriminate method that kills many non-target species, namely mesocarnivores and scavengers [11,36]. Nevertheless, both techniques will be used in Alberta, as recommended by government biologists and university-based academics advocating for this caribou fencing program [38,39]. Finally, Alberta trappers use mostly killing neck snares to capture wolves [40]. Manual and power killing neck snares are inadequate to consistently and quickly render canids unconscious. Furthermore, they are non-selective, and they impact seriously on the welfare of non-target animals [40]. It is worth mentioning that, as it was apparent with the wolf culling issue [41], the general public is deeply concerned about activities that place wildlife at risk and employ methods that fail to meet animal welfare standards [16]. In the 1980s and 1990s in the United States [42] and in Canada [43], the public and conservation groups agreed that non-selective wolf control programs were unacceptable.

## 6. Is there an Alternative Approach to the Proposed Predator-Free Fencing Program?

An elementary principle of wildlife conservation has always been the retention, protection, and restoration of large connected patches of critical habitats in a large scale connected network [44,45,46]. In this respect, Proulx [5] proposed an action plan where priority zones encompassing muskegs that are currently used by caribou, and a network of interconnecting movement corridors, would be protected from further disturbance, and where industrial activities would be closely monitored to minimize future habitat changes. Habitat restoration would involve re-establishing suitable vegetation including tamarack-black spruce stands in muskegs that are no longer used by caribou and on linear features that cross functional muskegs, and re-vegetating linear features that provide access to predators and people to high-quality muskeg habitats. Habitat protection and restoration, and the maintenance of caribou populations, could then be ensured without jeopardizing the integrity of wildlife communities. Such program recently received the endorsement of 18 wildlife professionals [47]. Importantly, a decade ago, an attempt to raise caribou in pens led the authors to conclude that “*penning is only effective if other land management and conservation strategies are implemented concurrently….. e.g., habitat condition will serve to benefit calf recruitment and survival [7]”.*

## 7. Discussion

The goal of contemporary wildlife biologists and conservationists is generally focused on accommodating human activity and occupancy while protecting biodiversity and the ecological functions and processes that maintain that diversity [48]. However, after nearly 40 years of research showing the negative impacts of fencing on wildlife communities and their environment, conservationists have largely abandoned fences [49] for the sake of ecosystem-based biodiversity conservation. 

According to the Director of Land and Environmental Planning North for Alberta Environment and Parks, the government’s proposed program would avoid the potential caribou population extirpation while ensuring there are enough healthy caribou to maintain a self-sustaining population in a range that would contain sufficient habitat [50]. However, on the basis of the failed results of a previous caribou rearing facility [7], an unsuccessful wolf culling program that did not result in an increase of caribou numbers [9], and no specific strategies to increase the amount of functional caribou habitat [3], we conclude that it is unlikely that the government’s proposed program will meet its objective. In our assessment, we argue that the Alberta Government’s predator-free and competitor-free fencing program will likely fail to (1) safeguard the long-term recovery of the LS caribou population; (2) produce animals that are representative of a wild free-ranging population; and (3) safeguard the welfare of wildlife communities and individuals. Knowing that an alternative program aimed at conserving and increasing habitat can be developed without jeopardizing the integrity of wildlife communities, we conclude that the government’s proposed fencing program is an ineffective conservation option. Furthermore, this has been recently recognized by the Director of Land and Environmental Planning North for Alberta Environment and Parks who stated that “the predator and alternate prey management program alone will not save caribou from extinction” [50].

The Aseniwuche Winewak Nation (AWN), located near the LS caribou range, expressed deep concerns that the caribou initiatives over the past decades have failed to take real action for caribou. They were deeply dismayed by the exclusion of Indigenous peoples from the Alberta Ministerial Task Force. Also, the AWN is cautious about using fencing on a large scale to protect caribou from predation, noting that there are many unanswered questions about the effects of such a fence on the local ecosystem [21].

Any future action for the recovery of the LS caribou population should be comprehensive and focused on biodiversity conservation and animal welfare. The proposed large-scale, predator-free and competitor-free fenced enclosure is anything but this. We believe that this fencing program has been largely conceived not with conservation as the real goal but as a politically expedient option to avoid taking meaningful action to conserve and restore woodland caribou habitat in ways that might even marginally impinge on whole scale intensive industrial expansion. All experts agree that widespread and immediate habitat restoration is the only real long-term solution for the Little Smoky caribou population, yet there seems to be no notable movement in this direction. That the proposed fencing of a caribou farm will include significant ongoing industrial activity (that may potentially consider voluntary slowing or stopping during the critical calving period) suggests that there is no meaningful buy-in from industry for this proposal and raises critical questions about how likely the proposed caribou farm will succeed. If the current and proposed actions do ultimately ‘save’ a small band of boreal woodland caribou over the immediate short term, but we completely lose the integrity of the ecosystem through even further modification of the landscape, collapse of food webs, and permanent further modification of wildlife population dynamics, we have accomplished nothing and have lost everything. We recommend that the international scientific community along with the general public and other groups object to the implementation of Alberta Government’s proposed flawed recovery program for the LS caribou population. 

## 8. Conclusions

We conclude that the Alberta Government’s proposed recovery program for the LS boreal woodland caribou will likely fail to safeguard the long-term future of this caribou population because it is only aimed at short-term ineffective strategies that have political appeal and are relatively cheap, but have no basis in science-based ecosystem management. The proposed plan does not include a long-term comprehensive habitat conservation program that will protect and interconnect muskegs that are used by caribou, and restore muskegs that are no longer used by caribou. While it is unlikely that the government’s proposed predator-free and competitor-free enclosure will safeguard the long-term recovery of the LS caribou population, the program will jeopardize wildlife communities inside and outside the fenced enclosure, and will cause harm to wild populations and individual animals. The rhetoric that predator control and caribou farming are the only options is based solely on the fact that no other options have been attempted. We conclude that the Alberta Government’s proposed program for the recovery of the LS caribou population is an ineffective conservation option.

## Appendix A

Excerpts from the Alberta Government’s Little Smoky caribou program [3] focused on the creation of a predator-free and competition-free 100 km^2^ fenced enclosure.

### *Habitat* *conservation*

Caribou habitat will be managed through the reduction of forest harvesting (≤ 498,500 m^3^/year within the Little Smoky caribou range), modifications to how oil and gas resources are managed involving voluntary activity rescheduling, protection from natural disturbances and coordinating industrial development to reduce footprint. Restoration of legacy seismic lines will begin immediately and new footprint will be minimized and mitigated.

### *Caribou fencing and wolf* *reduction program*

Alberta will construct a large (up to approximately 100 km^2^) fenced caribou rearing facility, to contain a suitable breeding population of caribou within the Little Smoky range. Periodically, young adult caribou will be released to the caribou population outside of the facility to contribute to population growth. The approach provides several potential benefits:

- Year-round protection for adult and young caribou from predation;
- Infrequent removal of predators from within the fenced area;
- Relatively large area protection, so caribou should require minimal supplemental feeding;
- Animals released as young adults should have reduced predation mortality rates; and
- The size and location of the facility will assist in it not contributing to negative impacts for the main caribou population remaining outside of the fenced area.

Alberta will continue to manage ungulate harvest levels to: (1) address increases in the productivity of moose, deer and elk which result from wolf population reductions; and (2) to reduce apparent competition between caribou and other prey species. The Government of Alberta will continue its existing wolf population management program in and adjacent to the Little Smoky Caribou Range.
